# Changes in Protease-Activated Receptor and Trypsin-1 Expression Are Involved in the Therapeutic Effect of Mg^2+^ Supplementation in Type 2 Diabetes-Induced Gastric Injury in Male Adult Rats

**DOI:** 10.1155/2023/5703718

**Published:** 2023-05-16

**Authors:** Nasrin Mehranfard, Hossein Rezazadeh, Nepton Soltani, Azadesadat Hosseini Dastgerdi, Mahtab Ghanbari Rad, Maedeh Ghasemi

**Affiliations:** ^1^Neurophysiology Research Center, Cellular and Molecular Medicine Research Institute, Urmia University of Medical Sciences, Urmia, Iran; ^2^Department of Physiology, School of Medicine, Isfahan University of Medical Sciences, Isfahan, Iran; ^3^Department of Preclinical Medicine, School of Medicine, Najafabad Branch, Islamic Azad University, Isfahan, Iran

## Abstract

**Purpose:**

Gastric inflammation is common and usually severe in patients with type 2 diabetes mellitus (T2DM). Evidence suggests protease-activated receptors (PARs) are a link between inflammation and gastrointestinal dysfunction. Given that magnesium (Mg^2+^) deficiency is a highly prevalent condition in T2DM patients, we assessed the therapeutic role of Mg^2+^ on the factors involved in gastric inflammation in T2DM.

**Methods:**

A rat model of T2DM gastropathy was established using a long-term high-fat diet + a low dose of streptozocin. Twenty-four rats were divided into control, T2DM , T2DM + insulin (positive control), and T2DM + Mg^2+^ groups. At the end of 2-month therapies, changes in the expression of gastric trypsin-1, PAR1, PAR2, PAR3, PI3K/Akt, and COX-2 proteins were measured by western blot. Hematoxylin and eosin and Masson's trichrome staining were used to detect gastric mucosal injury and fibrosis.

**Results:**

The expression of trypsin-1, PAR1, PAR2, PAR3, and COX-2 increased in diabetes, and Mg^2+^/insulin treatment strongly decreased their expression. The PI3K/p-Akt significantly decreased in T2DM, and treatment with Mg^2+^/insulin improved PI3K in T2DM rats. Staining of the gastric antrum tissue of the insulin/Mg^2+^-treated T2DM rats showed a significantly minimal mucosal and fibrotic injury compared with those of rats from the T2DM group.

**Conclusion:**

Mg^2+^ supplement, comparable to insulin, via decreasing PARs expression, mitigating COX-2 activity, and decreasing collagen deposition could exert a potent gastroprotective effect against inflammation, ulcer, and fibrotic development in T2DM patients.

## 1. Introduction

Prolonged diabetes mellitus results in a wide variety of gastrointestinal (GI) dysfunctions such as gastropathy [1–3]. Magnesium (Mg^2+^) is the second-most prevalent intracellular cation, and its deficiency was found to be associated with the risk of developing metabolic syndrome and type 2 diabetes mellitus (T2DM) [4]. Diabetic gastropathy is characterized by a number of gastric neuromuscular dysfunctions such as abnormalities in gastric tone, myoelectrical activity, and contractility [1], and Mg^2+^ has been shown to be essential for normal muscle and neurological function [5]. Moreover, both Mg^2+^ deficiency [6, 7] and diabetic gastropathy [1, 8–12] are associated with an increase in inflammatory responses and the production of free radicals. Additionally, Mg^2+^ supplementation was found to decrease inflammatory responses and improve metabolic status in many clinical trials of diabetes [13, 14]. These data suggest that Mg^2+^ may improve the management of diabetic gastropathy via targeting inflammatory pathways.

Among molecular targets, the involvement of protease-activated receptors (PARs) in the development of chronic inflammatory diseases has been abundantly documented [15, 16]. PARs belong to the family of G-protein-coupled receptors and include four types PAR1, PAR2, PAR3, and PAR4. PARs are abundant in the GI tract and are activated by a variety of endogenous serine proteases including trypsin, mast cell *β*-tryptase, matriptase, several kallikreins, and pharmacologically by synthetic compounds [10]. Specifically, PAR1 and PAR2 directly mediate several inflammatory responses and contribute to the pathogenesis of diseases [17]. Recently, PARs activation has been reported to aggravate disease progression in diabetes. For example, PAR-2 activation has been shown to potentiate diabetic nephropathy [18]. Also, Mitsui et al. reported that PAR1 and PAR2 cooperatively contribute to the pathogenesis of diabetic kidney disease and suggested that dual inhibition of both PAR1 and PAR2 may provide a promising novel therapeutic approach for treating the patients with diabetic kidney disease [19].

Several studies have demonstrated that PARs and cyclooxygenase-2 (COX-2) signaling pathways may be interrelated [20, 21], and that both PAR activation and increased COX-2 are associated with poor prognosis in patients with diabetes mellitus [22–25]. COX-2 is a principal mediator of inflammatory pathways, and its increased levels have been shown in several GI diseases such as gastritis and gastric tumors [26]. COX-2 has been suggested to be essential for the maintenance of gastric mucosal integrity [27], and its selective inhibitors have been found to delay the healing of ulcers and erosions in rodents [28, 29]. COX-2 was also found to contribute to the glucagon-induced delay in gastric emptying and has been suggested to be involved in diabetic gastroparesis [25]. The activation of the phosphatidylinositol 3′ kinase (PI3K)/Akt has been reported to be needed for the upregulation of COX-2 expression [30]. Further studies proposed that activation of the PI3K/Akt signaling pathway is a principal mechanism underlying the anti-inflammatory impacts of Mg^2+^ [31]. Additionally, an abnormality in the PI3K/Akt signaling pathway has been reported to be associated with T2DM [32]. However, whether Mg^2+^ has beneficial impacts and the possible mechanisms on the diabetic gastropathy remain unclear. This research aimed to examine the impacts of long-term oral Mg^2+^ supplementation on the improvement of the diabetes-induced proinflammatory responses by assessing the induction of gastric trypsin, PARs, PI3K/Akt, and COX-2.

## 2. Materials and Methods

Twenty-four male Wistar rats (180–250 g) were housed in the medical school of Isfahan University of Medical Sciences. Experimental producers follow the USA National Institute of Health (NIH, 1985) guideline and were approved by the animal care and ethics committee of this university (approval no: IR.MUI.RESEARCH.REC.1398.832, 12 March 2020). All animals had access to a standard housing cage with ad libitum water and food under controlled temperature (22 ± 2°C) and 12 : 12 light-dark cycles with 50 ± 5% humidity during the experiment. The T2DM rat model was developed according to our previous research [33].

### 2.1. Experimental Procedures

**Figure 1:**
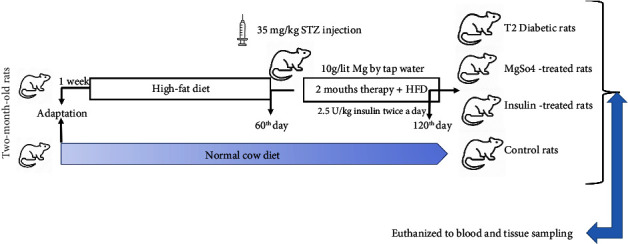
Schematic depiction of timelines for the experimental procedures. HFD: high-fat diet, STZ: Streptozotocin.

After stabilization for a week, rats were randomly divided into 2 groups: control group fed with a normal pellet diet and the T2DM rat model fed with a high-fat diet (HFD) for 8 weeks followed by a single injection of 35 mg/kg streptozocin (STZ) (Sigma Aldrich, Germany). The content of HFD (1 kg) comprises NPD powder (365 g), lard (310 g), casein (250 g), cholesterol (10 g), vitamin and mineral mix (60 g), DL-methionine (3 g), Yee-sac powder (1 g), and sodium chloride (1 g). Fasting blood glucose (FBG) was measured 3 days after diabetic induction, and rats with more than or equal to 250 mg/kg blood glucose were considered as the T2DM rat model. Then, diabetic rats were randomly divided into 3 different groups: T2DM rats, T2DM rats treated with 2.5 U/kg twice a day of insulin (T2DM + Ins), and T2DM rats treated with 10 g/lit MgSO_4_ (T2DM + Mg^2+^) by tap water via drinking water and usual HFD for 2 months. Insulin therapy was used as a positive control because it is often an important part of the T2DM therapy. The blood glucose levels of all groups were evaluated every week. In Figure 1, we show a schematic depiction of time line for the experimental procedures.

### 2.2. Biochemical Analysis

After all the elucidated processes, overnight fasted rats were euthanized by CO_2_, and the blood samples were collected. The stomach was separated and rinsed with cold phosphate-buffered saline (PBS) (pH = 7.4), and immediately stored at −80°C. The serum levels of FBG were measured by an automated analyzer using enzymatic methods (Pars Azmun, Iran).

### 2.3. Western Blot Analysis

To investigate alterations in the expression of trypsin-1, PAR1, 2 and 3, COX-2, PI3K, Akt, and p-Akt proteins, rat gastric antrum tissues were dissected out after completing the 120-day T2DM + treatment procedure (*n* = 6 for each group). The dissected antrum sections were rinsed with cold PBS and stored at −80°C for later use. The proteins were extracted with cold lysis buffer, protease inhibitor and phosphatase inhibitor cocktail. The proteins were isolated on 8% or 10% SDS-polyacrylamide gels and then transferred from the gels to polyvinylidene difluoride membranes (Bio-Rad, Hercules, CA, USA). The membranes were blocked with 5% skim milk in tris-buffered saline with Tween (TBST) and incubated with primary antibodies for trypsin-1, PAR1, 2, and 3, COX-2, PI3K, phosphorylated Akt (p-Akt), *β*-actin (SANTA CRUZ BIOTECHNOLOGY, USA), and Akt (Elabscience, USA) at 4°C overnight, and then with the appropriate HRP-conjugated secondary antibodies for 1 hour at room temperature. Western blot detection was performed using enhanced chemiluminescence, and analysis was conducted with two repetitions. The protein band was quantified by densitometry using Image J software (National Institutes of Health, Bethesda, MD, USA). The protein expression was normalized to *β*-actin and then to the control group.

### 2.4. Tissue Histopathology Index

For histopathological assessment, samples of the gastric antrum were taken, rinsed with ice-cold saline (4°C), and then fixed in a buffered formalin solution (10% formaldehyde). Paraffin-embedded sections of gastric antrum tissue (5 *μ*m thick) were prepared and stained with hematoxylin and eosin (H&E).Also, gastric fibrotic alterations were evaluated by Masson's trichrome staining. Each microscopic evaluation was performed by the pathologist, who was blind to the groups.

## 3. Statistical Analysis

The results were reported as mean ± S.E.M, with *p* < 0.05 that was considered significant. Differences between groups were evaluated by one-way ANOVA with the Tukey post hoc test. Protein expression analyses were evaluated by relative quantification to the control group.

## 4. Results

### 4.1. Changes in Fasting Blood Glucose in T2DM Rats following Long-Term Administration of Insulin or Mg^2+^ Supplement

**Figure 2:**
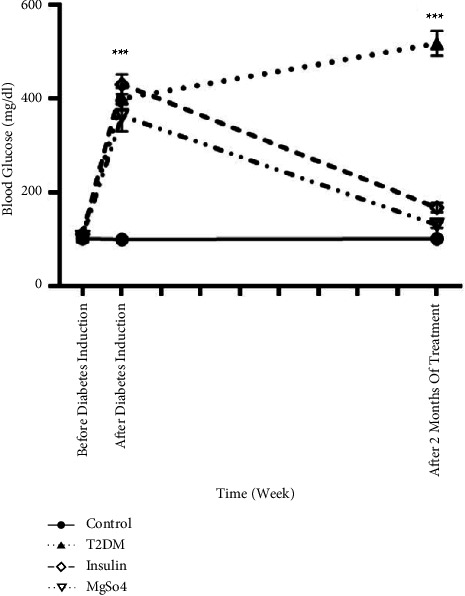
Effects of long-term administration of insulin and Mg^2+^ on blood glucose concentrations in T2DM rats (*n* = 6). The data are expressed as the mean ± SEM, ^*∗∗∗*^*p* < 0.001 vs. control.

Figure 2 shows FBG on the first day (before T2DM induction), third day (after T2DM induction), and sixtieth day of treatment. FBG was noticeably (*p* < 0.001) higher in T2DM rats than in control rats during the experiment. Long-term treatment with insulin or Mg^2+^ supplement significantly reduced the FBG of diabetic rats (*p* < 0.001) in comparison with the T2DM rats.

### 4.2. Changes in Body Weight in T2DM Rats following Long-Term Administration of Insulin or Mg^2+^ Supplement

**Figure 3:**
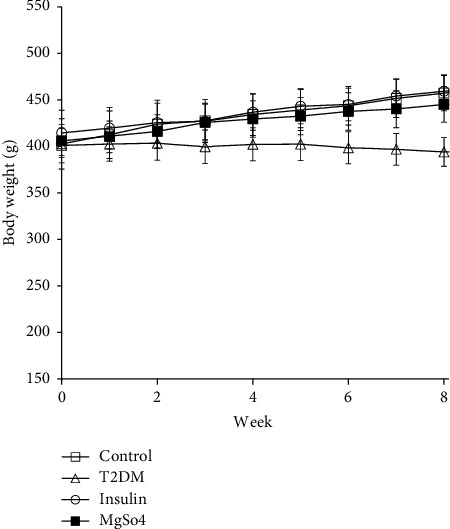
Weekly body weight measurements (g/groups). The data are expressed as the mean ± SEM, ^*∗∗*^*p* < 0.01 vs. control, ^$^*p* < 0.05 vs. T2DM.

The body weight of the animals was measured weekly after the induction of diabetes. The statistical results showed that the weight of the animals receiving insulin and Mg^2+^ significantly increased compared to the untreated diabetic group, while the largest weight increase was related to the nondiabetic control animal group (Figure 3) (*p* < 0.01).

### 4.3. Changes in the Expression of Trypsin-1, PARs, and COX-2 Proteins in Gastric Antrum of T2DM Rats following Long-Term Administration of Insulin or Mg^2+^ Supplement

**Figure 4:**
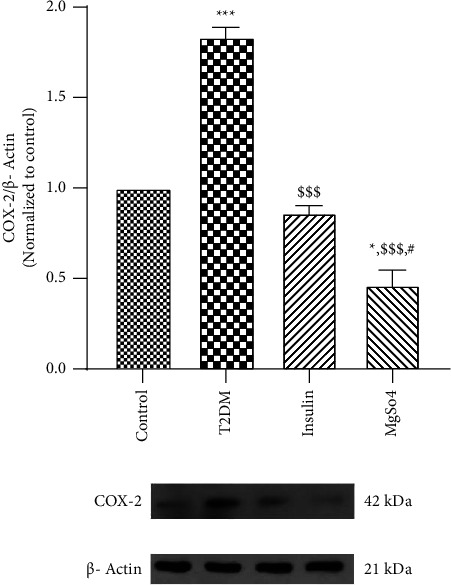
Gastric antrum expression of COX-2 protein. The data are expressed as the mean ± SEM, ^*∗*^*p* < 0.05 and ^*∗∗∗*^*p* < 0.001 vs. control; ^$$$^*p* < 0.001 vs. T2DM; ^#^*p* < 0.05 vs. insulin treatment.

As shown in Figure 4, COX-2 level was markedly increased (about 2 folds, *p* < 0.001) in the gastric antrum of T2DM rats. In the insulin-treated diabetic rats, COX-2 expression levels indicated no significant change in comparison with the control group. Interestingly, COX-2 expression in Mg^2+^-treated diabetic rats was decreased sharply compared to the control and diabetes groups (*p* < 0.05 and *p* < 0.001, respectively).

**Figure 5:**
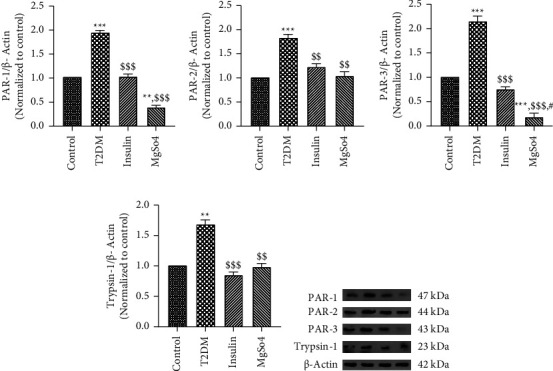
Gastric antrum expression of PARs (PAR-1, PAR-2, and PAR-3) and trypsin-1 proteins. The data are expressed as the mean ± SEM, ^*∗*^*p* < 0.05, ^*∗∗*^*p* < 0.01, ^*∗∗∗*^*p* < 0.001 vs. control; ^$$^*p* < 0.01 and ^$$$^*p* < 0.001 vs. T2DM; ^#^*p* < 0.05 vs. insulin treatment.

The expression level of PARs proteins (PAR-1, PAR-2, and PAR-3) in normal and diabetic gastric antrum tissues was detected by western immunoblotting. We found that PAR-1, PAR-2, and PAR-3 expression levels in the diabetic antrum tissue were markedly higher than those in the normal antrum tissue (*p* < 0.001, Figures 5(a)–5(d)). Compared to the diabetic rats, treatment with insulin or Mg^2+^ meaningfully reduced the expression of PARs proteins in the diabetic antrum tissue (PAR-1: *p* < 0.001, PAR-2: *p* < 0.01 and PAR-3: *p* < 0.001). Interestingly, PAR-1 and PAR-3 levels were markedly reduced in the Mg^2+^-treated diabetic rats compared to the control group (*p* < 0.001 and *p* < 0.01, respectively).

As shown in Figure 5(d), trypsin-1 showed significantly higher expression (1.6-fold, *p* < 0.01) in the gastric antrum of the diabetic rat compared with the controls (*p* < 0.01). Data analysis demonstrated that long-term treatment with insulin or Mg^2+^ effectively reduced the expression of trypsin-1 in the antrum of T2DM rats (*p* < 0.001 and *p* < 0.01, respectively).

### 4.4. Alterations in Expression of PI3K/Akt Pathway Proteins in the Gastric Antrum of T2DM Rats following Long-Term Administration of Insulin or Mg^2+^ Supplement

**Figure 6:**
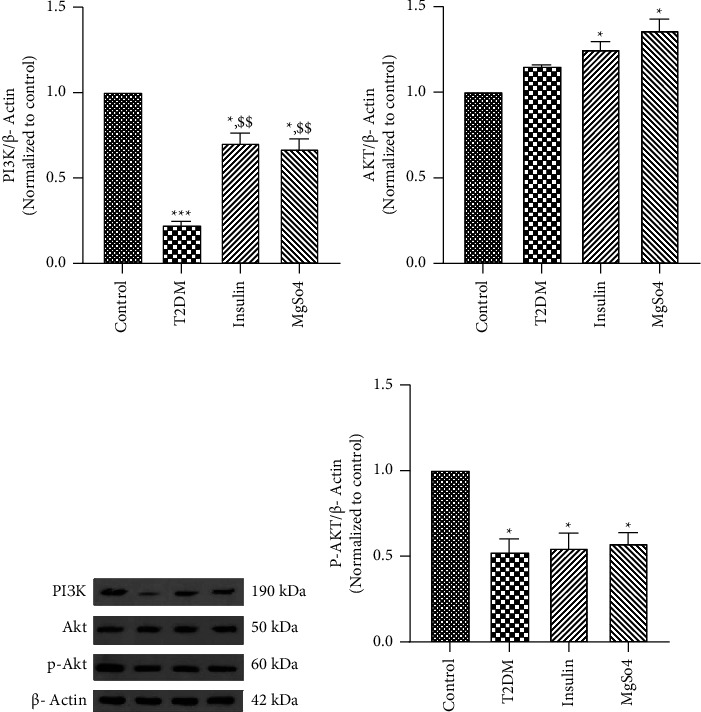
Gastric antrum expression of PI3K, Akt and p-Akt proteins. The data are expressed as the mean ± SEM, ^*∗*^*p* < 0.05, and ^*∗∗∗*^*p* < 0.001 vs. control; ^$$^*p* < 0.01 vs. T2DM.

As mentioned earlier, PI3K/Akt signaling is known to be essential for cell survival and metabolism; therefore, we evaluated the expression of three key proteins of this signal pathway PI3K, Akt, and p-Akt. Western blot indicated a significant decrease in PI3K and p-Akt expression in the gastric antrum of the T2DM rats, without any changes in Akt protein, compared to the control rats (Figures 6(a)–6(c), *p* < 0.001 and *p* < 0.5, respectively). The target proteins showed different changes in the treated groups. PI3K protein showed significantly higher expression in the insulin or Mg^2+^-treated T2DM rats in comparison with the nontreated T2DM rats (*p* < 0.01). No significant alterations in the levels of both Akt and p-Akt were observed in the treated T2DM rats compared to the nontreated T2DM rats, while the treated diabetic rats indicated a significant reduction in the level of p-Akt protein compared to the control rats (*p* < 0.05).

### 4.5. Histopathological Evaluation

**Figure 7:**
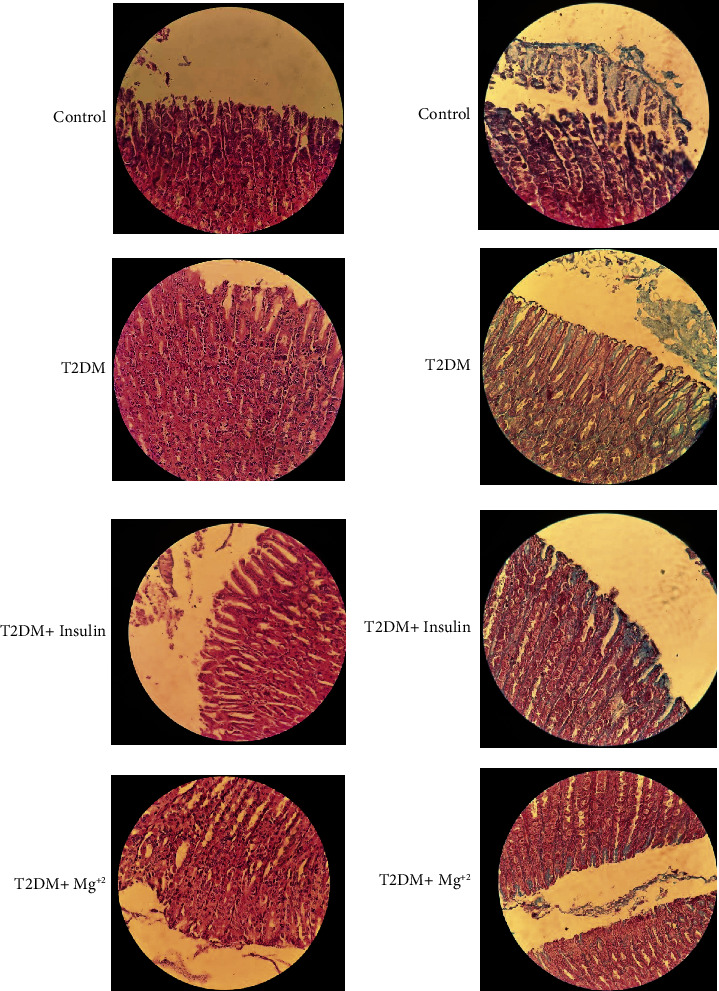
Histochemical staining of rat gastric antrum. (a) Hematoxylin and eosin staining; (b) Masson's trichrome staining in control, T2DM, and diabetic rats treated with Mg^2+^ and insulin (H&E and Masson's trichrome 40X).

H&E staining of control samples revealed normal gastric mucosal layers resting on the submucosa, with normal gastric pits and regular mucin-secreting epithelium. The gastric gland is seen in a uniform and regular manner with columnar cells containing basal nuclei and uniform cytoplasm (Figure 7(a), H&E, 40X). In Masson's staining (Figure 7(b), 40X), the mucin material is seen in blue color in the gastric pits, and the cells of the wall of the pits are in a regular row, and their cytoplasm is seen in blue color. Mucin secretions are also normal.

In the long-term T2DM group, the changes that are expected to be observed in the mucosa layer include its susceptibility to stomach ulcers. As shown in Figure 7(a) , the changes in this group are clear. The nuclei are hyperchromic, the cytoplasm has disappeared in some areas, and the epithelium of the stomach has also fallen and is destroyed and prone to damage. The effect of diabetes on the shape and size of the cells and nuclei was observed (Figure 7(a), H&E, 40X). Mucin in the diabetic rats also decreased compared to the control. Less mucin makes the stomach susceptible to ulcers.

Significant changes were observed in the T2DM + insulin group compared to the T2DM group. The pits and cytoplasm are regular. No changes were observed in the mucus compared to the control group. Masson's staining revealed a lower amount of mucin compared to the control group (Figure 7(b), 40X). Moreover, there is no change in number, thickness, and shape of the covering cells of the gastric glands compared to the control group. These changes showed an improvement of the gastric mucosa layer in the diabetic group treated with insulin.

In the T2DM + Mg^2+^ group, almost all the effects of the treatment have been observed. Gland cells are normal and regular, although compared to the control group, a little irregularity of the nuclei and a decrease in mucin are observed. The return of mucin from the lumen to the cells is also seen in this group (Figure 7(b), 40X).

**Figure 8:**
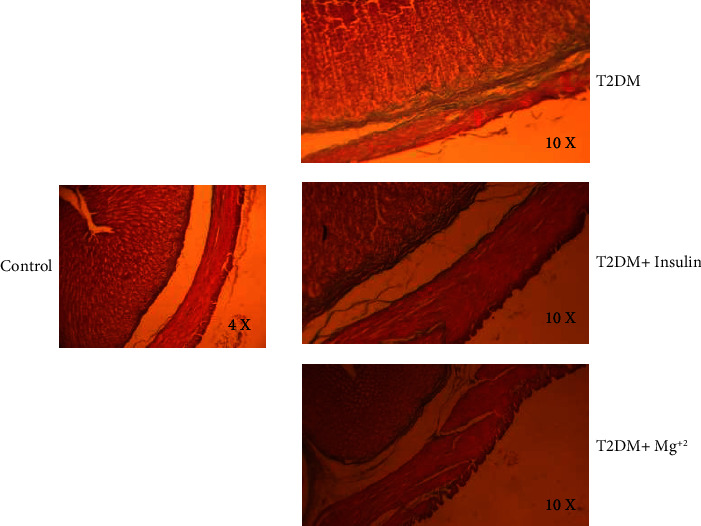
Morphology of gastric antrum muscle by Masson's trichrome staining in the different groups. Control group (10X); T2DM group (40X); T2DM + insulin treatment group (40X); and T2DM + Mg^2+^ treatment group (40X).

Moreover, Masson's trichrome staining of samples revealed the occurrence of fibrosis in gastric wall layers (Figure 8; 10X & 40X). In Masson's trichrome staining, the collagen fibers were stained blue in submucosa and muscularis mucosae and externa layers. The collagen fibers were rare in the control group. In the T2DM group, the collagen fibers were increased obviously in the muscularis mucosae, submucosa, and muscularis externa layers (Figure 8, 40X). Treated with insulin, the collagen fibers decreased slightly in all layers. In the T2DM + Mg^2+^ group, collagen fibers were sparsely distributed; just muscularis mucosae collagen was dyed a little blue (Figure 8, 40X).

## 5. Discussion

In the current study, we assessed the therapeutic effect of Mg^2+^ supplement and insulin on T2DM-induced inflammatory responses and tissue injury in the rat gastric antrum by assessing changes in trypsin-1, PAR receptors, PI3K/Akt/p-Akt, and COX-2 proteins levels and the histopathological index.

Gastropathy is a serious complication for diabetic patients, but its etiology is unclear. PARs play a principal role in the interactions between endothelial cells, clotting proteases of platelets, and smooth muscle cells in the vessel wall to control hemostasis, vascular tone and barrier function, cell adhesion, and inflammatory responses [15, 34]. The PARs are expressed by cells in every area of the GI tract [35, 36]. Previous studies have separately indicated the expression of PAR 1, 2, and 3 receptors in the stomach [37–39]. Consistent with the studies, our western blot analysis of the normal preparation revealed the occurrence of PAR1, 2, and 3 proteins in the rat gastric antrum. Although the expression of PARs has been evidenced in the GI tract, the biological significance of PARs is not yet fully understood. Overexpression of PARs (gene/protein) has been shown in different tissues of diabetic patients and animal models. For example, induction of diabetes was associated with enhanced levels of PARs in the aorta [40, 41], different parts of the kidney [19, 42], and in human vascular smooth muscle cells [17, 43]. These studies suggest that increased PARs activation may be involved in the pathophysiology of diabetic complications and inflammation in diabetic nephropathy, retinopathy, and cardiovascular disease. Accordingly, PARs inhibitors or genetic deletion of PARs have been shown to ameliorate diabetic-induced tissue damage [19, 44–46]. Mitsui et al. showed that suppression of PAR1 and PAR2 additively improved glomerulosclerosis and inflammation in diabetic kidney disease in mice [19]. They also demonstrated that PAR1 and PAR2 activation by agonists contributes to vascular inflammation in diabetes. Another study showed that induction of diabetes in mice lacking PAR2 (PAR2−/−) did not influence endothelial function of aortic segments compared to nondiabetic PAR2−/− mice, and PAR2 agonist induced endothelial dysfunction in the aorta of normal mice but not in that of PAR2−/− mice [44]. Lack of PAR2 was also found to improve increased levels of fibrosis- or proinflammatory-related markers in diabetic Akita mice [45]. Furthermore, an increase in the expression of PAR-4 has been demonstrated in diabetic human and murine vessels, and that the diabetic mice deficient in PAR-4 were protected from excessive vessel remodeling [46]. These alterations are in line with the present results obtained by the diabetic rats and their treatment with insulin and especially Mg^2+^ supplement. Our results revealed that both treatments decreased the elevated levels of PARs and the mucosal destruction and fibrosis in the diabetic rats, and particularly, Mg^2+^ caused a sharp downregulation in the expression of PAR 1 and 3. The reduced expression of PARs following both Mg^2+^ and insulin treatments in the present study may be associated with improved diabetes-induced gastric damage. In our study, increased PAR levels were associated with an increase in the level of the inflammatory mediator COX-2 in the gastric antrum of diabetic-treated groups. Previous studies have also demonstrated that inflammatory mediators regulate the expression of PAR2 and PAR4 [15, 47]. These data propose a reciprocal relationship between the PAR activity and inflammatory mediators. Our presented changes in the levels of PARs and COX-2 are similar to the outcomes of the studies that used selective receptor antagonists and gene deletion in inflammation or diabetes states. Both PAR1 and PAR2 activation by selective agonists are known to enhance the release of cytokines and inflammatory mediators. This suggests that the blockade of PARs might improve diabetes-induced GI dysfunction, in part, by decreasing inflammatory responses by affecting COX-2 expression.

In the current study, we also evaluated the expression of trypsin-1, as a PAR-activating protease. Trypsin or trypsin-like enzymes have been detected in some normal tissues and tumor cells [48–50]. We revealed that trypsin-1 is expressed in the normal rat gastric antrum. The diabetic rats showed an increase in the expression of trypsin-1, resulting in the activation of PARs. In addition to trypsin-1, some studies reported that the plasma levels and activity of thrombin, a main serine protease, are increased in diabetes, which may be another potent activator for PAR receptors. PAR-1 and PAR-2 were also found to elicit the secretion of pepsinogen, a serine protease, in the stomach [51, 52]. An exact mechanism for this secretory impact has yet to be clarified, although the expression of PAR-2 on chief cells proposes that the stimulatory effect of PARs on pepsinogen secretion may be direct [51, 52]. Hence, under chronic diabetes conditions, serine proteases, PARs, and inflammatory mediators appear to be in a positive strengthening cycle, resulting in more tissue damage. Consequently, this pathway may be a therapeutic target for reducing gastric tissue damage induced by diabetes. Considering the anti-inflammatory effect of insulin, Hyun et al. showed that insulin treatment decreases the PAR2-mediated inflammation in an insulin-deficient murine type 1 diabetes model [50]. Moreover, the therapeutic potent effect of Mg^2+^ on the inhibition of endotoxin-induced upregulation of inflammatory responses has been shown in previous studies [14, 53, 54]. Clinical studies have also demonstrated that an improvement in glycemic control can reduce inflammatory responses [55, 56]. Rezazadeh et al. proved that Mg^2+^ through increasing the expression of IRS1, Akt, and GLUT4 genes helps to improve insulin resistance and glycemic control in high-fat diet diabetic rats [33]. Our data also confirmed that Mg^2+^ supplement and insulin therapy improve positively the blood glucose in diabetic rats. The improvement of glycemic control may contribute partly and directly to the gastroprotective role of Mg^2+^ or insulin. Taken together, our results revealed an increase in the expression of trypsin-1, PAR1, 2, 3, and COX-2 in diabetes, and treatment with insulin, and especially Mg^2+^, markedly inhibited the expression of COX-2, trypsin protease-1, and PARs in the diabetic rats.

Growing evidence indicates that the PI3K/Akt signaling is needed for normal cellular metabolism, cell cycle progression, cell survival, coordinating defense mechanisms in innate immunity, and apoptosis [57, 58]. In addition, an imbalance in PI3K/Akt signaling has been associated with obesity, T2DM, and their complications [32]. Under normal conditions, growth factors, such as epidermal growth factor and insulin-like growth factor, influence PI3K/Akt signaling through their respective receptors [59]. When excessive energy intake occurs, as in our high-fat model of diabetes, excess circulating free fatty acids and hyperglycemia impair the PI3K/Akt signal [32]. Zhang et al.'s study developed a diabetic rat model to assess the timing of the occurrence of diabetic gastroparesis [60]. In this study, alterations in PI3K and p-Akt levels were observed during the establishment of the diabetic gastroparesis model, with an initial increase and then a decrease at 6 weeks. A reduction in the level of the PI3K/Akt/p-Akt pathway proteins was also found following our model of chronic T2DM. In our study, the expression of the PI3K and p-Akt proteins decreased following 8 weeks of diabetes induction. Consistent with our results, Zhang et al. showed a reduction in the activity of PI3K-Akt with the prolonged duration of diabetes [60]. In addition, there are some reports that demonstrate that trypsin and PAR2 agonists enhance the secretion of cytokines and chemokines via either mitogen-activated protein kinase (MAPK) or PI3K pathways [16, 61]. Therefore, manipulation of PI3K/Akt signaling, and its downstream molecules may be hopeful therapeutic targets for the treatment of T2DM.

Insulin has been shown to regulate cellular metabolism through an effect on the PI3K/Akt signaling pathway. Furthermore, previous studies revealed that increasing PI3K/Akt activity underlies the anti-inflammatory impacts of Mg^2+^. In our study, insulin or Mg^2+^ therapy resulted in the upregulation of PI3K/Akt without any significant changes in p-Akt expression in the diabetic rats. Due to the fact that phosphorylation is required for maximum activation of Akt, in order to evaluate the effectiveness of both treatments for diabetes-induced gastric injury, it is essential to assess downstream proteins of the PI3K/Akt pathway.

Evidence from human and experimental models of T2DM has implicated gastric mucosal destruction and fibrosis as strong pathological contributors. Fibrosis can increase gastric stiffness and decrease compliance, which results in gastric motility dysfunction. The major component of fibrosis is the excessive deposition of collagen fibers in tissue. Our histopathological findings also demonstrated that the intensity of fibrosis was significantly higher in the T2DM group than in the control group. In contrast to the T2DM group, the collagen fibers were significantly decreased in Mg^2+^ treated T2DM groups. These results suggest that Mg^2+^exhibits gastroprotective effect by inhibiting the excessive production of gastric collagen and alleviating fibrosis in diabetic rats.

Decreased muscle mass associated with decreased muscle strength and/or function, is a chronic complication of T2DM. It has been determined that T2DM-induced gastropathy is partly due to the damage and reduction of the mass of the stomach muscles, which have trouble clearing things out of them. In a recent study, glycemic control and use of insulin were significantly associated with an increase in skeletal muscle mass in patients with T2DM [62].

Considering that the previous findings strongly show that Mg^2+^ supplementation has the ability to improve insulin resistance [33, 63], it seems that the improvement of muscle mass and function in T2DM is partly due to the improvement of the insulin signaling pathway. In line with the histopathology findings, the weight results in the present study also confirm the improving effect of Mg^2+^ and insulin on body muscles, including the smooth muscles of the digestive system (stomach).

Together, our data suggest a potential role of serine proteases in diabetic gastropathy. Our study reveals an inhibitory effect of insulin or Mg^2+^ supplement on the inflammatory response of COX-2 that is probably triggered by PARs upregulation in diabetes. Hence, increasing PI3K/Akt activity and reducing the activation of COX-2 may underlie the potent anti-inflammatory impact of the treatment with insulin or Mg^2+^ in the T2DM. Thus, in the setting of insulin resistance in T2DM, the decreased insulin signaling and enhanced PARs activation may increase gastric tissue damage due to reduced PI3K/Akt activity and enhanced inflammatory responses.

## Data Availability

The data used in this study are made available upon reasonable request to the corresponding author.
